# Intact context memory performance in adults with autism spectrum disorder

**DOI:** 10.1038/s41598-021-99898-2

**Published:** 2021-10-14

**Authors:** Sidni A. Justus, Patrick S. Powell, Audrey Duarte

**Affiliations:** School of Psychology, Georgia Institute of Technology, 654 Cherry St. NW, Atlanta, GA 30332-0170 USA

**Keywords:** Psychology, Human behaviour

## Abstract

Research on memory in autism spectrum disorder (ASD) finds increased difficulty encoding contextual associations in episodic memory and suggests executive dysfunction (e.g., selective attention, cognitive flexibility) and deficient metacognitive monitoring as potential contributing factors. Findings from our lab suggest that age-related impairments in selective attention contribute to those in context memory accuracy and older adults tended to show dependence in context memory accuracy between relevant and irrelevant context details (i.e., hyper-binding). Using an aging framework, we tested the effects of selective attention on context memory in a sample of 23 adults with ASD and 23 typically developed adults. Participants studied grayscale objects flanked by two types of contexts (color, scene) on opposing sides and were told to attend to only one object-context relationship, ignoring the other context. At test, participants made object and context recognition decisions and judgment of confidence decisions allowing for an evaluation of context memory performance, hyper-binding, and metacognitive performance for context judgments in a single task. Results showed that adults with ASD performed similarly to typically developed adults on all measures. These findings suggest that context memory performance is not always disrupted in adults with ASD, even when demands on selective attention are high. We discuss the need for continued research to evaluate episodic memory in a wider variety of adults with ASD.

## Introduction

Autism spectrum disorder (ASD) is characterized by socio-communicative impairments and restricted or stereotypical patterns of interests and behavior^1^. It is becoming increasingly clear that memory deficits may be present in populations with neurodevelopmental disorders, including adults with ASD^2^. The memory profile of individuals with ASD suggests that deficits are not homogenous across all memory subsystems^3^ or uniform across individuals with ASD^4,5^. Episodic memory is one area where research has predominantly found evidence for mild impairment in individuals with high-functioning autism (HFA) or Asperger’s syndrome^2,6^.

Episodic memory is long-term memory for our daily life experiences. Episodic memory includes encoding of contextual features (location, time of event) associated with the event itself, which allow us to distinguish events from one another at retrieval. A number of studies have demonstrated intact item memory among adults with ASD but deficits in memory for contextual details associated with those items or events^7–9^. For example, some studies using “remember/know” procedures in individuals with ASD show that they may *know* that they have seen or heard something before but are less able to *remember* the contextual details of those memories^10–12^. Recent meta-analyses have pointed out the variability in performance in episodic memory studies of ASD, highlighting the heterogeneity in multiple areas, including sampling characteristics, outcome measures (e.g., words, pictures, stories), and retrieval demands (e.g., recall, recognition)^6^. For example, contextual details embedded within episodic memory can be differentiated based on the extent to which they are stimulus-bound (i.e., intrinsic or belonging to the study object) or spatiotemporal (i.e., extrinsic or not part of the study object)^13^. While intrinsic features are thought to be processed automatically and remembered on the basis of familiarity, extrinsic features require more attentional resources to encode and necessitate recollection to be recovered^14^. Results are mixed with regards to whether type of stimuli and context feature impact memory deficits in individuals with ASD. For example, in some studies, individuals with ASD have shown greater context memory impairments for spatiotemporal or extrinsic features compared to stimulus-bound or intrinsic features^15^ but others have found intact performance for spatiotemporal information^16,17^. Also, consistent with the executive dysfunction hypothesis of symptomatology in ASD^18^, episodic memory impairments also tend to be greater when tasks are highly complex and/or minimal support is provided to facilitate encoding or retrieval^19^. For example, episodic memory is particularly poor for individuals with ASD compared to participants with typical development (TD) on tests of free recall^20–22^, and often intact in tests of cued recall or recognition^2,7,23^. The benefits of task support highlight the influence that cognitive control demands have on memory performance in individuals with ASD. This pattern of results is not surprising as studies have suggested a stronger relationship between executive function and episodic recollection in ASD compared to neurotypical controls^24–26^.

Numerous parallels between the memory profiles of ASD and that of typical aging have led researchers to utilize an “aging analogy” to explain memory impairments sometimes seen in ASD^11^. For example, typical aging features increased relational memory difficulties^27^ similar to that seen in ASD^3,7^ compared to item memory. Furthermore, similar difficulties in free recall but intact recognition and increased reliance on task support are seen in typical aging literature^28^. Relative to memory for items, episodic memory for item features is disproportionately dependent upon frontally-mediated executive control processes^29^. Emergent research has suggested that context memory accuracy can be improved in typical aging when orienting instructions or tasks are presented during encoding and direct attention to task-relevant associations [e.g., “Is this color (context) likely for this object (item)?”] compared to when attention is directed to a single item or non-contextual features^30–35^. However, daily life involves multiple context features and our ability to remember certain details likely depends on how well we ignore others. Thus, deficits in inhibitory control would likely impede context memory performance in the presence of task-irrelevant distractors. Reductions in selective attention during encoding could lead to the formation of memories that include both task-relevant and irrelevant details, a process known as *hyper-binding*^36,37^*.* Consistent with this idea and the age-related inhibitory control hypothesis^38^, we^39^ have shown that TD older adults, compared to young TD adults, show reduced context memory accuracy and increased binding of distracting contextual information (i.e., ‘hyper-binding’)^36,37^. Inhibitory control deficits, including difficulties with distractor interference, are also common in ASD^40,41^. Therefore, following an aging analogy, one might expect adults with ASD to also show a reduction in context memory performance because of increased binding of the relevant and irrelevant contextual details.

Poorer episodic memory performance in ASD may also be at least partly accounted for by metacognitive monitoring or metamemory difficulties^42–44^. Metamemory includes the knowledge about our own memory (i.e., how it works and how we can maximize it through strategic strategy use)^45,46^. Selective attention is suggested to indirectly affect metacognitive abilities by increasing the amount of perceptual evidence available, which lowers the decision bound, boosting visual and metacognitive awareness^47^. Well-known tests of metacognitive ability involve asking participants not only to answer questions about recently studied material or stored knowledge but also to report confidence in answers provided [e.g., judgment of confidence (JOC), feeling of knowing (FOK), or judgment of learning (JOL) tasks]. If metacognitive monitoring is good, performance judgments should accurately discriminate between correct and incorrect answers (indicating confidence-accuracy correspondence). Results from ASD metamemory studies remain scarce and inconclusive with some finding no differences compared to TD in metacognitive accuracy (Ref.^48–50^, but see Ref.^51^). In contrast, others find differences but only in certain age groups (e.g., children but not adults^52^, but see Ref.^53^), or certain types of memory judgments (e.g., episodic not semantic)^54^. Studies assessing retrospective JOC for contextual or source details in adult samples have also found conflicting results, with some finding impaired metamemory in ASD^55^ and others finding no group differences in metacognitive monitoring but subtle differences in metacognitive monitoring^56^.

The few studies that have found differences between ASD and TD suggest potential impairments in metacognition for ASD^51,53,55,57^. For example, Grainger et al.^53^ found less accurate FOK judgments in adults with ASD but greater self-reported metacognitive abilities than TD adults. These findings suggest the potential for diminished correspondence between metacognitive monitoring and memory accuracy despite high self-confidence in metacognition in ASD. However, ASD metamemory studies frequently use correlation coefficients between accuracy and confidence (e.g., Gamma coefficient)^58^, which risks confounding confidence judgment sensitivity with response biases^59^. Specifically, differences in participants’ confidence-accuracy correlation coefficients could result from an overall likelihood to give high confidence endorsements for their responses rather than a true reflection of a difference in sensitivity. Maniscalco and Lau’s^59^ meta-dprime (*metad’*) is grounded in signal detection theory (SDT)^60^. It allows for separation of sensitivity (i.e., how well confidence ratings discriminate between a participant’s correct/incorrect responses) and response bias (i.e., how likely a participant is to endorse responses with high vs. low confidence) of metacognitive performance. It also remains to be seen how hyper-binding of task-irrelevant distractors might affect both context memory and memory confidence in ASD. Increased memory load due to hyper-binding might weaken the memory trace, resulting in a reliance on familiarity and diminished confidence for context memory judgments as confidence is typically low for guess-based judgments^61^.

Taken together, existing literature suggests that episodic memory may be particularly impaired in ASD when demands on executive function and/or metamemory are high. In our lab, we have designed a novel selective attention context memory task that places heavy demands on executive function and has been previously used to evaluate item and contextual memory in neurotypical aging^39,62,63^. To our knowledge, no studies have investigated hyper-binding in ASD. Using an aging framework to investigate episodic memory in ASD, we would predict increased hyper-binding in ASD compared to TD. Further, our task asks participants to make judgments of confidence (JOC) at retrieval for both attended and unattended contexts. Metamemory is still an understudied area in ASD research and, to our knowledge, this is only the second to explore retrospective memory confidence for contextual-level details in adults with ASD^56^ and the first that uses *metad’* which separates response biases from sensitivity unlike commonly used Gamma correlations. When metacognitive differences have been seen in ASD compared to neurotypical controls, results suggest diminished confidence-accuracy correspondence^51–53^ which has also been seen in healthy aging^64–66^. However, mixed results and minimal preliminary findings from adult samples make it currently unclear whether difficulties observed in contextual metamemory performance would be seen in adults with ASD in the present study.

To our knowledge, no other studies have explored context memory, hyper-binding, and metamemory for contextual details in the same sample of adults with ASD compared to TD adults. It is not currently known how selectively attending to one contextual feature while attempting to ignore another feature influences both contextual memory and metamemory performances in ASD compared to TD adults. Previous work has primarily tested context memory for intrinsic features (e.g., color of the line drawing^8^) or spatiotemporal extrinsic details (e.g., order or location of presented word on screen^17^). However, the present task features visual target and distractor contexts presented extrinsically (i.e., alongside) a central greyscale object. Extrinsic details are thought to require more attention and effortful encoding compared to the automatic processing of intrinsic details^14^. Individuals with ASD have been shown to have problems with encoding episodically bound visual details due to problems engaging the attentional processes that would facilitate successful encoding^67^. The present study adds to the existent literature because to our knowledge, existing studies have used relational orienting instructions for extrinsic features (e.g., RiSE paradigm^68^) but only for one item-context pair. By adding a second, distractor context we increased task complexity in hopes that our study could better evaluate the ability for adults with ASD to engage attentional processes to facilitate successful relational encoding and context memory for extrinsic details. The present task also allows for a direct manipulation of executive function, which, while not the sole explanation for episodic memory(see Ref.^69^ for review), has been shown to be implicated to a greater extent compared to TD^26,70^. If selective attention is reduced in ASD, an increase in hyper-binding and reduction in context memory may be observed. Further, we predict that increased hyper-binding would result in less vivid memories and, in turn, would reduce one’s confidence in memory decisions (i.e., reduced metamemory performance). For example, in our previous study of neurotypical aging^39^, older adults showed decreased context memory performance facilitated by increased hyper-binding and reduced memory confidence, even for correct memory decisions. We aimed to establish whether these abilities are impaired in ASD in a way that aligns with an aging analogy. In order to do so, we compared context memory performance in a sample of adults with ASD (ages 18 to 58 years) to a sample of age, gender, and education matched typically developing adults. Specifically, we use a complex context memory task which we have previously employed in younger and older adults without ASD^39,62,63^ to investigate whether adults with ASD demonstrate 1) impaired item or context memory, 2) increased patterns of hyper-binding, and 3) less retrospective confidence-accuracy correspondence compared to adults without ASD. If ASD involves impairments similar to that seen in neurotypical aging, we would expect to see significant decreases in episodic memory performance and increased hyper-binding among adults with ASD compared to TD controls. ASD metamemory performance may also show less confidence-accuracy correspondence similar to that seen in neurotypical aging, but this was not assumed a priori based on existing literature.

## Methods

### Participants

Twenty-three participants (17 males, 6 females) with ASD (18–58 years, *M* = 30.2, *SD* = 12.3) and 23 TD participants (14 males, 9 females, 18–57 years, *M* = 28.7, *SD* = 13.1) were included in this study. A subset of the data from the TD sample was included in prior publications^39,62,63^. Participants were right-handed, native English speakers with normal or corrected-to-normal visual acuity and normal color vision. No participants reported psychiatric or neurological disorders, vascular disease, or use of medications affecting the nervous system. All participants were recruited through the Georgia Institute of Technology Psychology participant pool, advertisements on public transportation, newspapers, word of mouth, and referrals from programs in the greater metro-Atlanta area serving adults with ASD. All participants were compensated either $15/hour or with extra credit for their psychology courses. Study protocols were approved by the Georgia Institute of Technology Institutional Review Board (IRB) and were carried out in accordance with the approved guidelines and regulations. We obtained written consent from all participants on an IRB-approved consent form prior to participation in our study.

Participants completed a standardized neuropsychological battery to provide a broader cognitive profile of our participants (see Table 1). The neuropsychological battery included subtests from the Memory Assessment Scale (MAS)^71^ and the Halsted-Reitan Neuropsychological Test Battery (HRNB)^72^. MAS included immediate and delayed list recall which were chosen as orthogonal measures of non-pictorial episodic memory, allowing for a broader investigation of memory performance in the current sample of adults with and without ASD. HRNB included phonemic letter fluency (‘FAS’ version of Controlled Oral Word Association Test^73^) which is a commonly used neuropsychological measure of phonemic verbal fluency that is conceptualized as a measure of executive function^74^. Trails-Making Test (TMT^72^) Trails A (processing speed) and B (processing speed + cognitive flexibility) subtests were also chosen as well-validated and widely used measures of executive function that are less language-focused. Given prior literature in adults with ASD^75,76^, we predicted lower performance on these tests in ASD than TD adults. ASD diagnosis was confirmed by clinical interview using the Autism Diagnostic Observation Schedule-2 (ADOS-2)^77^ using the following inclusion criteria: 1) prior diagnosis of ASD and 2) met the ADOS-2 diagnostic cut-off score (> 7) and Social Responsiveness Scale-2 (SRS-2)^78^ diagnostic cut-off (> 60). For this study, we initially considered all available ASD participants’ data (*n* = 25). Two participants were excluded from the ASD sample, one for not meeting criteria based on the ADOS-2 and one for failure to complete the behavioral task and difficulty completing neuropsychological tests (i.e., severe difficulty grasping task instructions). Each participant with ASD (*n* = 23) was then matched to a TD control with regard to age [*t*(44) = 0.41, *p* = 0.687, Cohen’s *d* = 0.12], gender [*p* = 0.530, Fisher’s exact test],and education [*t*(39) = 0.80, *p* = 0.427, Cohen’s *d* = 0.25]. Participant demographics and neuropsychological test scores are summarized in Table 1.

**Table 1:** Participant demographics.

Measure	ASD (17M/6F)^+^			TD (14M/9F)^+^		
	*M*	*SD*	Range	*M*	*SD*	Range
Age^+^	30.2	12.3	18–58	28.7	13.1	18–57
Education^+^	14.4	1.7	12–18	14.8	1.8	12–18
Letter fluency	14	4	6–21	14.2	3.9	9.3–27
List recall (immediate)	9.3	2.3	2–12	10.2	1.3	7–12
List recall (delayed)	9.8*	2.0	6–12	11*	1.4	7–12
Trails A (in sec.)	32.2	19.9	8–90	26.1	8.0	13–44
Trails B (in sec.)	83*	55.8	9–251	53.3*	22.9	26–128
**ADOS-2 module 4**^a^						
Communication	4	1.7	2–8	–	–	–
Reciprocal social interaction	8	2.1	5–13	–	–	–
Combined total	12	3.4	7–20	–	–	–
**SRS-2 (T-score)**^b^						
Social awareness	61	8.7	44–78	–	–	–
Social cognition	66	8.7	48–81	–	–	–
Social communication	71	7.9	52–86	–	–	–
Social motivation	69	10.4	42–81	–	–	–
Restricted interests behavior	74	9.3	57–90	–	–	–
Combined total	71	7.0	60–85	–	–	–

*Note.* ^+^ indicates variables used for matching. * indicates significant group difference (*p* < 0.05). ^a^Autism diagnostic observation schedule-2 (ADOS-2), module 4. ^b^Social responsiveness scale-2, adult form self-report (SRS-2).

### Materials and design

The task utilized a pool of 432 grayscale images of objects collected from the Hemera Technologies Photo-Object DVDs and Google Images. Each image featured a single, nameable object centrally fixed on a white background. On opposite sides (left/right) of the object were 1 of 3 possible colored squares (red, brown, or green) and 1 of 3 possible scenes (studio, city, or island). The positions of these flanking contexts (color/scene) were counterbalanced so that they were presented an equal number of times on the right and left sides of the central object. Object and context images spanned a maximum horizontal and vertical visual angle of approximately 3°. During encoding, 288 objects were presented, and participants were instructed to direct their attention to one of the two attended contexts (either the colored square or the scene) on each trial. At retrieval, all 288 previously presented objects (i.e., “old trials”) were presented along with 144 new objects that had not been previously studied. Old/new status for objects was also counterbalanced across subjects.

### Procedure

**Figure 1:**
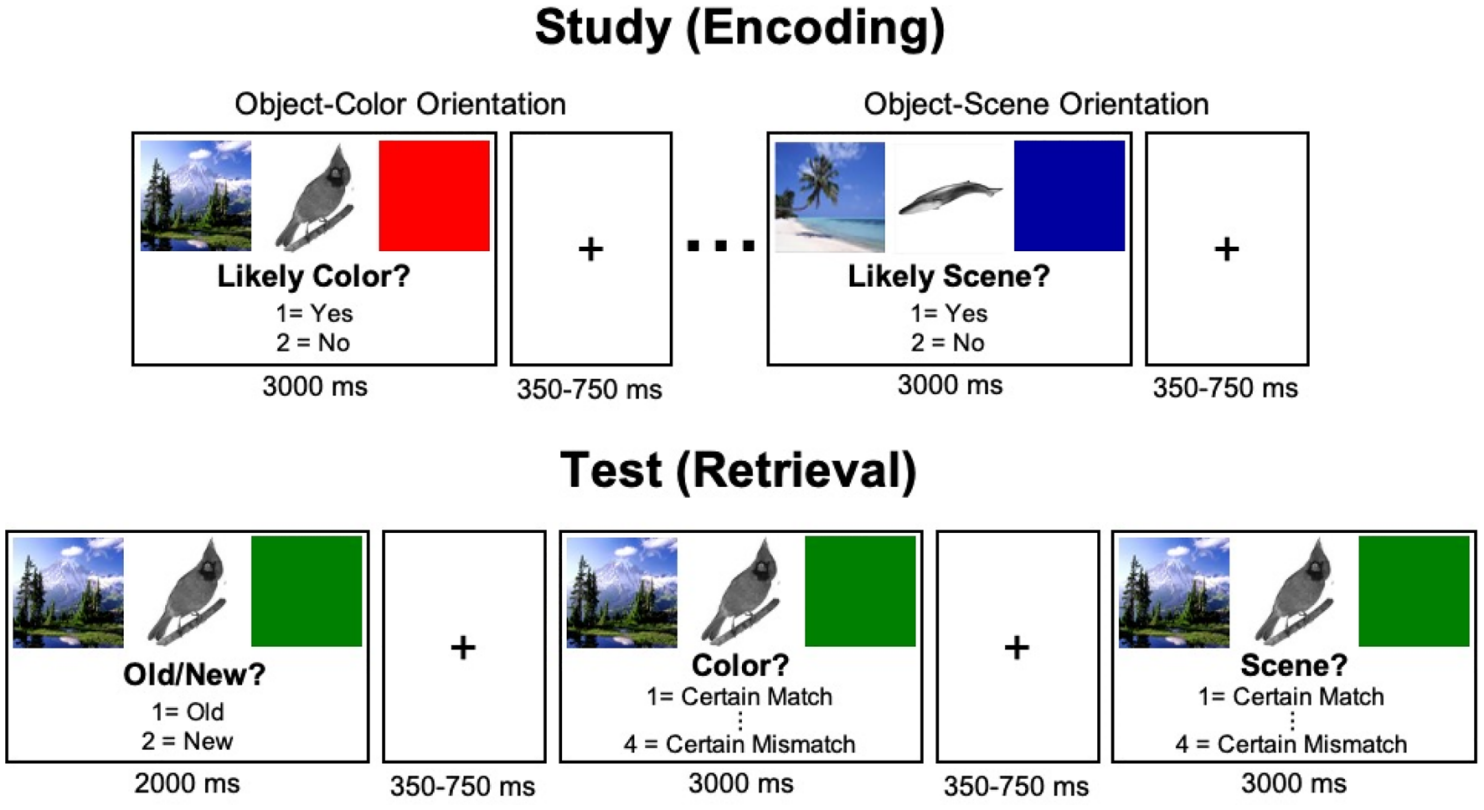
Experimental design.

Participants were informed about study purposes and procedures and written consent was obtained. The procedure was split into four study blocks (288 trials, 72 trials per block) and four test blocks (432 trials, 108 trials per block). Participants completed all four study then all four test blocks. All participants were given instructions and a brief practice of study (average ~ 5 min) and test blocks before beginning the experiment. Practice was repeated until participants demonstrated an understanding of the procedure. Figure 1 illustrates the study (encoding) and test (retrieval) structure and timing.

**Figure 2:**
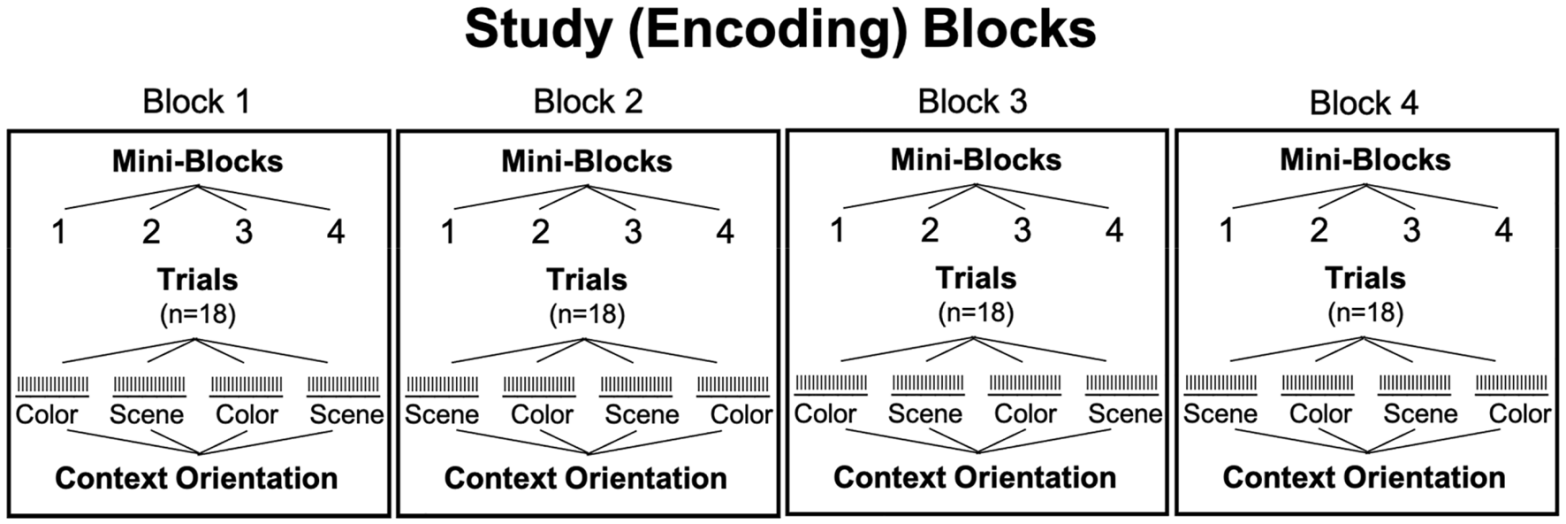
Mini-block design of study phase. Four mini-blocks per study block, 18 trials per mini-block.

During study blocks, participants were asked to make subjective (yes/no) judgments about the relationship between the grayscale object and *either* the scene (i.e., “Is this object likely to appear in this scene?”) *or* the colored square (i.e., “Is this color likely for the object?”). Both verbal and written instructions instructed participants to orient their attention to one context and ignore the other context for each trial. Participants responded with one of two keypresses to indicate “yes” or “no.” Study blocks were each divided into four mini-blocks of 18 trials, each for a total of 72 trials per block (Fig. 2). These mini-blocks were designed to both orient the participant to which context they should pay attention to on the subsequent trials, but also to reduce the task demands of having to switch back-and-forth between judging the two different contexts (color, scene) which previous evidence has suggested disrupts memory performance in older adults^79^. Piloting determined that the blocking procedure was necessary to ensure suitable performance across participants of all ages in this study. Prior to beginning each mini-block, participants were prompted with a question, “Likely color?” or “Likely scene?” to inform them of which judgment to make. These prompts remained on the screen underneath the images on each trial. The order of which context judgment was prompted first (i.e., object-color or object-scene) was counterbalanced across participants.

During test blocks, participants were presented with both old and new objects. Similar to the study blocks, each object was flanked on opposite sides (left/right) by a scene and colored square. For old objects, the color/scene contexts were located on the same side of the object as they had been during study. Participants were first asked to respond via keypress whether the object was “old” or “new.” If they responded “new,” the next trial began after 2000 ms. If they responded “old,” they were then asked to make two additional judgments about the context features (i.e., one about the scene, one about the colored square). For these judgments, participants decided whether the context presented with the object matched the context that had been presented with that object during encoding. Participants responded using 1 of 4 keypresses to indicate scaled context match confidence judgments ranging from 1 (certain match) to 4 (certain mismatch). Again, the order of which context judgment was prompted first (i.e., object-color match, object-scene match) was counterbalanced across participants. Trials were designed that for “old” trials, objects were presented equally with (1) both color and scene matching those presented during study, (2) only scene matching, (3) only color matching, and 4) neither color nor scene matching.

### Analysis

Statistical analyses were performed using IBM SPSS 27 software^80^. Correlation analyses were used to explore relationships between neuropsychological tests and task performance. Analyses of variance (ANOVA) and analyses of covariance (ANCOVA) were used to explore differences in memory and metamemory performance within and between groups and controlling for age, respectively. Item recognition accuracy was estimated using Signal Detection Theory (*d’prime*) measure of discriminability: z (hit rate)—z (false alarm rate). Context memory accuracy was also computed as *d’prime* for attended and unattended context features separately using the following formula: *d′* = z (proportion of “match” responses to contexts that matched those presented at encoding)—z (proportion of “match” responses to contexts that mismatched those shown at encoding).

In order to directly examine hyper-binding of attended and unattended context features in each group, we calculated conditional probabilities similar to Uncapher et al.^81^. Specifically, the probability of correctly endorsing the attended context given that the unattended context was correctly endorsed was calculated as [p(Both correct)/[p(Both correct) + p(Unattended only correct)]. The probability of correctly endorsing the attended context given the unattended context was incorrect was calculated as [p(Attended only correct)/[p(Attended only correct) + p(Neither correct)]. Likewise, the probability of correctly endorsing the unattended context given that the attended context was correct was calculated as [p(Both correct)/[p(Both correct) + p(Attended only correct)]. Finally, the probability of correctly endorsing the unattended context given that the attended context was incorrect was calculated as [p(Unattended only correct)/[p(Unattended only correct) + p(Neither correct)]. A hyper-binding index was computed by subtracting the conditional probability of correctly endorsing the attended context given that the unattended context was correctly endorsed was calculated as [p(Both correct)/[p(Both correct) + p(Unattended only correct)] by the conditional probability of correctly endorsing the attended context given the unattended context was incorrect, [p(Attended only correct)/[p(Attended only correct) + p(Neither correct)]. Positive values indicate a greater likelihood of correctly recognizing both the target and distractor (i.e., hyper-binding).

For each trial to which participants responded ‘old’ (item recognition), they then decided whether each context (attended, unattended) matched or mismatched the context presented with the object at encoding. Embedded within this second decision was also a decision regarding confidence (high/low) in context memory judgments (i.e., participants chose either high confidence context match, low confidence context match, low confidence context mismatch, high confidence context mismatch). Metamemory performance was estimated using Signal Detection Theory (*meta*-*d’prime*)^59^ measure of discriminability: z (high confidence hit rate)—z (high confidence false alarm rate).

For all analyses, we evaluated whether conditions were met for applying parametric tests by inspecting box plots for outliers and testing for violations of normality (Shapiro–Wilk’s test *p* < 0.05) and homoscedasticity (Levene’s test *p* < 0.05). In any instances where outliers or violations of normality were found, we conducted analyses again after removing outliers. In all cases, we observed no change in statistical significance for any of the results reported below. Thus, we chose to report parametric results that include full data for all measures. Where analysis of variance (ANOVA) and analysis of covariance (ANCOVA) were used, effect sizes were calculated using partial eta-squared (*η*_*p*_^2^), where *η*_*p*_^2^ = 0.01, *η*_*p*_^2^ = 0.06, *η*_*p*_^2^ = 0.14 is considered small, moderate, or large effect size, respectively^82^. Where *t* tests were used, effect sizes were calculated using *Cohen’s d*, where d = 0.2, d = 0.5, d = 0.8 is considered small, medium, or large effect size, respectively^82^. Where non-significant main effects or interactions with Group (TD vs. ASD) were observed, Bayes Factor^83^ (null/alternative; BF_01_) were computed where < 0.33 or > 3 are considered noteworthy. Bayesian analyses were conducted using JASP 0.14.1 software^84^.

## Results

### Neuropsychological assessment results

Group demographics and neuropsychological test results are shown in Table 1. Scores for the MAS and HRNB neuropsychological battery subtests were missing for one ASD participant due to failure to complete and were replaced with the ASD group mean for each subtest. Adults with ASD exhibited significantly lower performance as compared to TD adults on Delayed List Recall [*t*(39) = 2.08 , *p* = 0.044, *Cohen’s d* = 0.61, equal variances not assumed, Levene’s test was significant] and Trails B, [*t*(44) = 2.36 , *p* = 0.023, *Cohen’s d* = 0.70]. There were no other significant group differences [*t*’s < 1.59, *p’s* > 0.12, *Cohen’s d’s* < 0.47].

### Object and context memory performance

Average group discriminability (*d’prime*) scores for item and context memory are shown in Table 2. Both groups showed above chance (0) performance for item recognition [*t*(22)’s > 7.52, *p*’s < 0.001, *Cohen’s d*’s > 1.57]. Item recognition did not differ between groups, [*t*(36) = 1.15, *p* = 0.26, *Cohen’s d* = 0.34, equal variances were not assumed, as Levene’s test was significant], suggesting comparable item memory performance between the adults with and without ASD Given the data, there is weak evidence in favor of an absence of a Group effect (BF_01_ = 2.01).

**Table 2:** Group averages in hit rate, false alarm rate, and discriminability (d’) for item and context memory.

Measure	Hit rate	False alarm rate	D’prime
I**tem recognition**			
ASD	0.69 (0.25); range 0.09–0.99	0.19 (0.28); range 0.01–0.99	1.78 (1.14); range − 0.13–3.93
TD	0.71 (0.15); range 0.43–0.96	0.08 (0.08); range 0.02–0.40	2.10 (0.68); range 0.66–3.31
**Attended context**			
ASD	0.74 (0.21); range 0.04–0.99	0.41 (0.22); range 0.07–0.99	0.96 (1.16); range − 1.86–3.76
TD	0.78 (0.14); range 0.32–0.97	0.41 (0.19); range 0.08–0.98	1.04 (0.67); range − 0.24–2.68
**Unattended context**			
ASD	0.54 (0.20); range 0.15–0.91	0.47 (0.19); range 0.08–0.84	0.20 (0.20); range -0.08–0.64
TD	0.56 (0.14); range 0.28–0.99	0.53 (0.16); range 0.30–0.95	0.12 (0.25); range − 0.20–1.03

*Note.* Mean (Standard Deviation).

Both groups also showed above chance (0) performance for both attended [*t*(22)’s > 3.98, *p*’s < 0.001, *Cohen’s d*’s > 0.83] and unattended [*t*(22)’s > 2.31, *p*’s < 0.031, *Cohen’s d*’s > 0.48] contexts. A Context (Attended vs. Unattended) × Group (TD vs. ASD) ANOVA revealed a main effect of Context, [*F*(1, 44) = 31.06, *p* < 0.001, *η*_*p*_^2^ = 0.41], but no main effect of Group, [*F*(1, 44) = 0.00, *p* = 0.99, *η*_*p*_^2^ = 0.00] or interaction between these factors, [*F*(1, 44) = 0.29, *p* = 0.59, *η*_*p*_^2^ = 0.01]. This suggests that the manipulation of attention during encoding was effective at enhancing context memory accuracy for the target context. Given the data, there is moderate evidence in favor of an absence of a main effect of Group (BF_01_ = 3.92) and an interaction between Group and Context (BF_01_ = 3.10). This pattern of effects remained when controlling for age: ANCOVA again revealed a main effect of Context, [*F*(1, 43) = 22.67, *p* < 0.001, *η*_*p*_^2^ = 0.35], no main effect of Group [*F*(1, 43) = 0.03, *p* = 0.86, *η*_*p*_^2^ = 0.00], and no Context × Group interaction [*F*(1, 43) = 0.18, *p* = 0.67, *η*_*p*_^2^ = 0.004].

**Figure 3:**
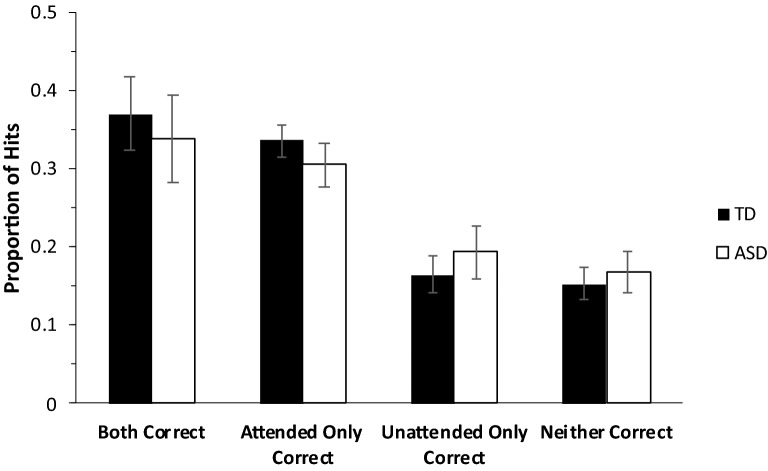
Proportions of hits associated with correct and incorrect judgments for both attended and unattended contexts. Note. Error bars depict 95% CI for mean.

Figure 3 depicts the mean proportion of correctly recognized objects (hits) for which the participant correctly judged both contexts (*Both correct* trials), only the target context (*Attended only correct)*, only the distractor context (*Unattended only correct*)*,* or only the item (i.e., neither context correctly judged; *Neither correct* trials). Consistent with the previous analyses, there were no group differences in performance [*t*(44)’s < 0.99, *p*’s > 0.33, *Cohen’s d*’s < 0.29]. Given the data, there is weak evidence in favor of an absence of an effect of Group on Attended correct (BF_01_ = 2.30) and Unattended correct (BF_01_ = 2.75) trials, and moderate evidence in favor of an absence of an effect of Group on Both correct (BF_01_ = 3.42) and Neither Correct (BF_01_ = 3.36) trials.

### Hyper-binding

All conditional probabilities used to examine hyper-binding are shown in Table 3. If either group is hyper-binding, they should show greater context accuracy for one feature if the other feature was also correctly recognized than if it was not recognized. If ASD adults are more likely to show hyper-binding, similar to that seen in older adults, they should show greater conditional dependence between attended and unattended context accuracy. To examine these possibilities, we conducted a Context (Attended vs. Unattended) × Accuracy of the other feature (Correct vs. Incorrect) × Group (TD vs. ASD) ANOVA which revealed a main effect of Context, [*F*(1, 44) = 72.79, *p* < 0.001, *η*_*p*_^2^ = 0.62] but no other significant main effects or interactions, [*F*(1, 44)’s < 3.50, *p’*s > 0.07]. Given the data, there is moderate evidence in favor of an absence of a main effect of Group (BF_01_ = 4.61) and the Accuracy of the other Feature*Group (BF_01_ = 3.20) and weak evidence in favor of the absence of the Context*Group interaction (BF_01_ = 2.47). Participants were more likely to judge attended than unattended contexts correctly, but the lack of Context × Accuracy interaction suggests no evidence of hyper-binding in either group. This pattern of effects remained when controlling for age: ANCOVA again revealed a main effect of Context, [*F*(1, 43) = 35.41, *p* < 0.001, *η*_*p*_^2^ = 0.45], but no other significant main effects or interactions, [*F*(1, 43)’s < 3.65, *p’*s > 0.06].

**Table 3:** Probabilities of correct context feature conditionalized on accuracy for the other context feature.

	ASD		TD	
	*M (SD)*	Range	*M (SD)*	Range
Attended correct if unattended correct	0.65 (0.19)	0.21–0.95	0.68 (0.12)	0.50–0.93
Unattended correct if attended correct	0.52 (0.12)	0.10–0.67	0.52 (0.07)	0.36–0.69
Attended correct if unattended incorrect	0.68 (0.13)	0.40–0.90	0.69 (0.10)	0.50–0.92
Unattended correct if attended incorrect	0.54 (0.06)	0.43–0.70	0.52 (0.08)	0.38–0.65

Follow-up hierarchical regression analysis was conducted to examine if the relationship between hyper-binding index as a predictor of context memory accuracy varied as a function of group. Attended context accuracy (*d’prime*) served as the dependent variable and Group (ASD vs. TD) and Hyper-binding Index were first entered (Model 1), followed by a Group × Hyper-binding Index interaction term (Model 2). Results indicated that Group (β = 0.04, *p* = 0.77) and Hyper-binding Index (β = 0.26, *p* = 0.08) were not significant predictors of attended context memory accuracy, [R^2^ change = 0.07 , *F*(2, 43) = 1.65, *p* = 0.20]. This remained true when the interaction of Group × Hyper-binding Index (β = − 0.12, *p* = 0.45) was added to the model [R^2^ change = 0.01, *F*(1, 42) = 0.57, *p* = 0.45].

### Metamemory performance

Average metamemory performance (*metad’)* scores for context memory and are shown in Table 4. We conducted a Context (Attended vs. Unattended) × Group (TD vs. ASD) ANOVA to evaluate differences in metacognitive performance (*metad’*). Results revealed a significant main effect of Context, [*F*(1, 44) = 40.41, *p* < 0.001, *η*_*p*_^2^ = 0.48] but no main effect of Group, [*F*(1, 44) = 0.05, *p* = 0.82, *η*_*p*_^2^ = 0.001] or interaction between these factors, [*F*(1, 44) = 0.82, *p* = 0.37, *η*_*p*_^2^ = 0.02]. Metacognitive performance was greater for attended than unattended contexts for all participants. Given the data, there is moderate evidence in favor of an absence of a main effect of Group (BF_01_ = 3.96) and weak evidence in favor of an absence of the Context*Group interaction (BF_01_ = 2.45). This pattern of effects remained when controlling for age: ANCOVA again revealed a main effect of Context, [*F*(1, 43) = 29.18, *p* < 0.001, *η*_*p*_^2^ = 0.40], no main effect of Group, [*F*(1, 43) = 0.29, *p* = 0.66, *η*_*p*_^2^ = 0.005] or interaction between these factors, [*F*(1, 43) = 0.66, *p* = 0.42, *η*_*p*_^2^ = 0.02].

**Table 4:** Group averages in metamemory performance (metad’) for context memory.

	ASD		TD	
	M (SD)	Range	M (SD)	Range
Attended context	0.99 (0.98)	− 1.15 to 2.58	1.08 (0.84)	− 0.29 to 2.83
Unattended context	0.25 (0.49)	− 0.97 to 1.44	0.09 (0.38)	− 0.66 to 0.87

Follow-up hierarchical regression analysis was conducted to examine the relationship between hyper-binding index as a predictor of attended context metamemory performance varied as a function of age and group. Attended context metamemory performance (*meta-d’*) served as the dependent variable and age was first entered (Model 1). Group (ASD vs. TD) and Hyper-binding Index were then entered (Model 2), followed by a Group × Hyper-binding Index interaction term (Model 3). Results indicated that age (β = − 0.48, *p* = 0.001) was a significant predictor of context metamemory performance, [R^2^ change = 0.23, *F*(1, 44) = 13.01, *p* = 0.001]. The addition of Group (β = 0.03, *p* = 0.85) and Hyper-binding Index (β = 0.26, *p* = 0.07) to the prediction of attended context metamemory did not lead to a statistically significant increase in R^2^, [R^2^ change = 0.06, *F*(2, 42) = 1.70, *p* = 0.196]. Introducing the Group × Hyper-binding Index (β = 0.04, *p* = 0.81) variable did not have a significant impact [R^2^ change = 0.001, *F*(1, 41) = 0.06, *p* = 0.81].

Metacognitive efficiency is calculated by observing the discrepancy between *d’* (accuracy) and *metad’* (metacognition) where *metad’*/*d’* = *1* indicates an ideal metacognition-performance relationship and the degree to which *metad’*/*d’* < *1* indicates the degree to which a participant is metacognitively inefficient^59^. We conducted a Context (Attended vs. Unattended) × Group (TD vs. ASD) ANOVA to evaluate differences in the ratio of *metad’/d’*. Results revealed no significant main effects or an interaction [*F*(1, 44)’s < 0.99, *p’s* > 0.33, *η*_*p*_^2^’s < 0.02]. This suggests that the confidence/accuracy relationship (metacognitive efficiency) does not significantly differ across contexts or groups. Given the data, there is moderate evidence in favor of an absence of a main effect of Group (BF_01_ = 3.01) and weak evidence in favor of an absence of a main effect of Context (BF_01_ = 2.77) and the Context*Group interaction (BF_01_ = 2.37). This pattern of effects remained when controlling for age: ANCOVA again revealed no significant main effects or interactions, [*F*(1, 43)’s < 2.85, *p’*s > 0.099, *η*_*p*_^2^’s < 0.06].

## Discussion

The goal of the present study was to investigate whether adults with ASD differed from typically developing adults in context memory accuracy, hyper-binding, or metamemory in a novel context memory task. Results revealed no significant group effects. Consistent with previous studies^4^, adults with and without ASD exhibited similar item-memory performance. However, the hypothesis that ASD would show decreased context memory performance despite intact item-level performance was not supported. For both groups, memory for contextual features was greater for the attended context compared to unattended contexts. Further, both groups were more likely to judge attended contexts compared to unattended contexts correctly, and this was not conditionally dependent on the accuracy of the other context, suggesting neither group was hyper-binding in this sample. Lastly, we found no group differences on measures of metamemory performance for context memory judgments.

These results are perhaps surprising given the literature documenting episodic memory impairments in ASD^2,4,85^. However, the present findings building upon previous studies from our lab^30,31,39,62,63^ and others^33–35^ showing that younger and older neurotypical adults can direct their attention toward task-relevant details in a way that supports context memory performance. In this study, this finding is extended to show that at least some adults with ASD, like TD adults, can successfully focus their attention on task-relevant details even in the presence of a task-irrelevant contextual distractor. It is possible that environmental support during encoding in this task (i.e., “is this object likely for this scene?”) attenuated context memory deficits that would have been seen in ASD. This aligns with findings from a recent fMRI study using the Relational and Item-Specific Encoding (RiSE^86^) protocol which features encoding support (e.g., asking whether an item in a pair could fit inside another) for pictorial associations found no group differences in relational memory performance, similar to the present study. Research has suggested that context memory accuracy can be improved when attention is directed to task-relevant associations compared to when attention is directed to a single item or non-contextual features^30–32,34,35^. It is possible that if given no direction on which object-context relationship to focus on that we might have seen effects more so aligned with theories of autism (e.g., executive dysfunction), suggesting that individuals with ASD have problems inhibiting distraction, which may lead to overlooking contextually significant relationships and paying too much attention to extraneous stimuli. The weak central coherence account^87,88^ of autism and differences in natural patterns of attention (see Ref.^89^ for review) support the idea that individuals with ASD would have problems binding important information in real-life scenarios where one has to decide which aspects of an event are or are not important^20^.

A non-mutually exclusive possibility is that the type of item-context association required in our task differs from those frequently employed in ASD studies of context memory. Individuals with ASD may have a differential ability to remember certain types of details of an episodic event. The variability in the literature might be explained by the degree to which different episodic tasks require individuals to encode and remember a variety of intrinsic and/or extrinsic details. For example, color can be an intrinsic surface feature (e.g., color of object^8^) or a feature of surrounding context (e.g., colored border encasing object^15^) manipulating same feature across intrinsic/extrinsic conditions. Intrinsic details are thought to be processed automatically as a byproduct of item processing whereas extrinsic details require greater attention and intentional coding^14^. Our task contexts are presented extrinsically and therefore we would have predicted larger impairments in adults with ASD compared to TD due to impairments in selective attention associated with ASD^40,41^. However, putting extrinsic context features into the focus of attention during encoding via explicit orienting instructions could have produced item-context binding in the present task. Not all context memory tasks include orienting instructions and those that have (i.e., RiSE paradigm mentioned above) also found no differences in memory performance for ASD compared to TD^68^. Research has supported the idea that context binding may be limited to features naturally in the immediate focus of attention (i.e., intrinsic, stimulus-bound details), but when intentional contextual learning is employed, intrinsic and extrinsic features may be processed similarly^90^. An episodic event relies on the associative binding of a variety of details including specific item features, multiple items, spatial locations, temporal contexts, and self-referential information. If relationships between facets of a given event are not adequately processed, context memory of any type would be impaired. Individuals with ASD may exhibit difficulties in ability remember extrinsic details due to difficulty spontaneously processing this information in the first place^67^. However, this does not imply that these individuals lack the capacity to process the relationships as evidenced by the comparable performance in ASD and TD groups in the present task under supported encoding conditions. Continued work examining context memory deficits in ASD as a function of the intrinsic/extrinsic nature of the to be remembered information is needed.

Another possible explanation for the inconsistencies between the current study and prior research demonstrating impaired context memory in ASD may relate to task differences at retrieval. Participants in the present study are re-presented with the previously seen pairs (e.g., object-context match) at test which may allow for reliance on familiarity-based recognition. Bowler et al.’s Task Support Hypothesis^7^ suggests that memory in ASD will be better on any task where test procedures include information about encoded material. Individuals with ASD show the greatest difficulties on tests of free recall compared to controls and group differences diminish on cued recall or recognition tasks^20^. However, recognition tasks with high demands on relational binding have found diminished recognition of object-context combinations despite intact recognition of individual context elements^8^ though findings have been mixed (e.g., Ref. ^9,97^). Possible explanations for inconsistent findings include the complex information processing model^19^ which suggests that memory in ASD reflects a general cognitive phenotype characterized by difficulties with organization, integration, and flexible use of information^98^. Tasks that provide support for reinstating context at retrieval require less reliance on effective cognitive organization strategies and therefore may not show impairment in ASD. Future studies could vary or remove orienting instructions or require participants to choose from all possible options the appropriate contexts (both attended and unattended) at test for items previously seen during encoding. Such methodological manipulations to minimize task support would allow for further exploration of the nuances of context memory impairments and the potential for hyper-binding in ASD.

Further, it should be noted that the neuropsychological test data (Table 1) suggest that some memory impairments are observed in the ASD group in this study, in contrast to what we observed in our context memory task. Specifically, ASD adults’ delayed list recall performance was lower than that of TD adults in this study. Furthermore, correlational analysis revealed relationships between Delayed List Recall and attended context memory *d’* in ASD, [*r*(21) = 0.38, *p* = 0.04] but not TD adults [*r*(21) = 0.16, *p* = 0.23]. This supports the idea that ASD adults’ episodic memory impairments may contribute to individual differences in context memory performance in our task, even though, on average, ASD and TD groups performed similarly. This discrepancy could be explained by the nature of these assessments and our task. As discussed earlier, one possibility is that the task support provided at both encoding and retrieval in our task may have attenuated episodic memory differences that would have otherwise been observed in this sample. It is also worth noting that these neuropsychological tests assess memory for words while our task assessed memory for pictures and associated colors and scenes. It is possible that the visual stimulus materials that we used resulted in enhanced memory, demonstrating a “picture superiority effect,” in which pictures are remembered better than word stimuli^99^. This effect is suggested to result from faster activation of semantic associations for pictures versus words, allowing for the generation of more robust and elaborative associations between stimuli^100^. A recent study by Ring and colleagues comparing episodic and semantic memory across verbal/visual and meaningful/meaningless materials showed better memory for visual and meaningful stimuli in both TD and ASD adults^101^. However, other studies have suggested that the picture superiority effect is less seen in ASD^2^. In particular, the additional elaborative encoding of pictures may not be seen in autism as studies have shown no benefit from semantic rather than solely perceptual-based encoding^88^. In the present study, a direct comparison of the object-scene and object-color pairs used in this study to word pairs would be needed to test a picture superiority effect hypothesis.

Although these results might indicate sparing of executive functioning in the ASD adults, it is important to note that they did show impaired performance on some neuropsychological tests (i.e., Trails B), indicating some executive dysfunction (Table 1). A significant negative correlation between Trails B response time, corrected for Trails A time (i.e., TMT B minus A^94,95^), with both attended context memory *d’* [*r*(21) = -0.46, p = 0.01] and the hyper-binding index in the ASD, [(*r*(21) = -0.50, *p* = 0.007] but not the TD group [*r*(21)’s < -0.31, *p*’s > 0.07]. Although hyper-binding was not greater in adults with ASD compared to TD like we predicted, it is maybe somewhat impaired in this group given the relationship with corrected Trails B. Lower executive function ability (i.e., flexibility) may result in difficulty shifting attention between our attend color and attend scene tasks. This would result in lower context memory accuracy and reduced shifting between the two extrinsic features (i.e., less hyper-binding). As mentioned previously, the lack of significant group differences in our task may be due to the supportive instructions provided that are not present in the neuropsychological tests. Nonetheless, executive dysfunction is evident and does appear to contribute to our context memory task. There is well-known heterogeneity in cognitive performance and behavior in ASD, leading some researchers to suggest potential subtyping within the ASD spectrum based on an executive function endophenotype^91^. Also, cross-sectional studies of childhood through adolescence have suggested that problems with resisting distractor interference may resolve with increasing age^92,93^ possibly indicating delayed rather than abnormal executive function development. However, research in ASD remains predominately focused on early childhood and adolescence and only recently has shifted to include studies of adults (see Ref.^94–96^ for review). Continued research is needed to determine if the age-related trajectory of executive functioning in ASD is similar to neurotypical aging. In neurotypical studies, context memory performance is associated with age-related decline and increased hyper-binding^36,39^. Future longitudinal studies involving larger, cognitively diverse samples of younger and older adults with ASD will be important for better understanding age-related versus ASD-related differences in episodic memory and hyper-binding^36,39^.

Adults with ASD also did not significantly differ from TD in a measure of metacognitive performance (*metad’*) or in the *metad’/d’* ratio which investigates confidence-accuracy correspondence. Metacognitive abilities are still understudied in ASD and findings to date have been inconclusive. Studies that report group differences suggest diminished confidence-accuracy correspondence for ASD, although this has not been conclusive in the literature (Ref.^51,52,54,57^, but see ^48–50^). Only one adult study had evaluated retrospective confidence for episodic memory finding no group differences similar to what we find here^56^. Other existing adult studies suggesting no group differences had methodological concerns (see Ref.^51^ for detailed evaluation) including inadequate age-matchings^50^ and incomplete reporting and interpretation of results (Ref.^52^ (exp. 2)). Existing ASD metamemory studies also utilize correlation coefficients (e.g., Gamma coefficient^58^) to assess metacognitive performance, which risks confounding the sensitivity and response biases. SDT Measures such as *metad-prime*^59^ used in the present study allow for separation of bias and sensitivity and should be widely utilized in future studies to investigate metacognitive monitoring in ASD.

The existing literature to date also varies significantly in how metamemory is defined and assessed in ASD. Different metamemory judgment tasks (JOC, FOK, JOL) used throughout the literature show only modest correlations with one another and may be tapping different processes^102–104^ which could explain inconclusive evidence regarding ASD metacognitive impairment. Further, studies of neurotypical populations have supported the idea that metamemory judgments are guided by recollection^105^ and contextual or source information^106^. Autism is associated with reduced recollection and reliance on familiarity for memory judgments^11,12^. Therefore, the degree to which recollection is important to task performance (i.e., familiarity cannot be relied on) would also determine the likelihood of reduced metamemory accuracy in ASD. In the present study, we cannot be sure whether our adults with ASD were relying more on familiarity or recollection for their memory and metamemory judgments. Future context memory tasks using Remember/Know judgments would be better suited to evaluating this.

Tasks that divide or direct attention at encoding could also affect the degree to which information can be bound into memory as the literature suggests encoding difficulties yield the episodic memory impairments seen in ASD (see Ref.^4^ for review). Therefore, the degree to which individuals with ASD experience difficulty binding important contextual information at encoding would also determine the likelihood of reduced metamemory accuracy in ASD (i.e., less contextual information available at retrieval on which to base metamemory judgments). In the present study, adults did not show reduced context memory or increased hyper-binding, suggesting successful encoding that may have been supported by orienting instructions. If we had seen overlap between the details of relevant and irrelevant contextual information (i.e., hyper-binding), then context memory and metamemory may have been negatively affected. The variability in the literature and current pattern of findings suggest that individuals with autism do not appear to suffer from a generalized metamemory deficit and that the task support hypothesis may also extend to underlying metamemory processes. It is possible that individuals with ASD are better able to monitor and regulate performance when environmental support aids memory performance, as in the current study.

It is important to acknowledge the present study’s limitations. One limitation is that we did not include a direct measure of inhibition. Though our hypothesis that adults with ASD would show increased hyper-binding compared to TD adults was not supported, increased hyper-binding is thought to stem from reduced selective attention due to underlying problems with inhibiting distractors^38^ which has been previously shown in ASD^40^. However, there are multiple facets to inhibitory control (see Ref.^107^ for discussion of taxonomy), including prepotent response inhibition, resistance to distraction, and resistance to proactive interference (PI). The neurotypical age-related inhibitory control hypothesis^38^ implicated in the aging analogy that we make in this paper here investigates the inhibition of PI. It is possible that inhibition of PI may be spared in the present ASD sample as evidenced by the lack of group differences in immediate list recall^108^. In the future, multiple measures of inhibitory control (e.g., inhibiting task-irrelevant visual information as in a Stroop task) should be included to better understand which aspects, if any, of inhibitory control are impacted in ASD. Further, we cannot truly know from this experiment what aspects of the items and contexts shown were focused on by participants. Research has shown individuals with ASD to exhibit atypical local/global processing compared to neurotypical controls. For example, individuals with ASD have been hypothesized to have enhanced local processing^88^, impaired global processing^109^, and/or a preference or bias towards local versus global information when given a choice^87^. In the future, an Embedded Figures test^110^, Block Design test^111^, or a Navon^112^ figure test could be used to evaluate local and global processing in all participants. Further, while we cannot truly know in our task if local aspects of the stimuli presented (e.g., a leaf on a palm tree in the island scene) were perceived more so than the Gestalt of the stimulus (i.e., island vs. city scene), future research could incorporate eye-tracking technology to investigate this possibility.

Another limitation of the present study is that we did not match on verbal IQ, which is commonly seen in ASD literature, as this data was not collected for the sample. However, we performed one-to-one matching considering age, education, and gender, and our resulting sample was comparable across groups on many of the neuropsychological measures that we collected (Table 1). Given the large variability often present in the cognitive profile of individuals with ASD, our results cannot be generalized to the entire population. Our ASD sample did vary considerably on the SRS-2 (total T-score range of 60–85 with 73% of sample scoring 66 + which indicates moderate to severe symptoms). This suggests that null group differences are unlikely to be due to the fact that the adults in our ASD group were predominately those with mild or sub-clinical ASD symptomology. Autism symptomatology lies on a continuum and a larger sample with the ability to examine severity as related to memory could be illuminating. Future studies including larger lifespan samples with diverse ASD symptomatology could better address questions regarding ‘aging in autism’ by investigating if the onset of changes in episodic memory performance seen in neurotypical aging is mirrored in ASD. It is also possible that the null effects including lack of group differences were due to a smaller sample size yielding insufficient power to detect smaller effects. However, as mentioned above, it is possible that our task provided sufficient support, diminishing group differences in context memory performance^7^. Despite these limitations, this study adds to the knowledge of episodic memory, contributing to heterogeneous and inconclusive literature regarding the extent of context memory impairments and metamemory abilities.

## Conclusion

To conclude, the present study, to our knowledge is the first to assess context memory, hyper-binding, and metacognitive performance for contextual details in a community sample of adults with ASD on a novel episodic memory task. Results show no evidence of differences in episodic memory or metacognitive performance between adults with and without ASD in our sample. It is possible that individuals with ASD show only minimal episodic memory or metamemory impairments compared to TD for context memory tasks involving visual stimuli. It is also possible that impairments exist (e.g., executive impairments indicated by neuropsychological measures) but that the present task offered enough support to overcome them or that deficient performance would not be seen until later adulthood due to age-related cognitive decline. Following the Task Support Hypothesis^7^, our findings suggest that support in the form of a relational orienting task at encoding may help adults with ASD overcome difficulties deploying relational memory processes for extrinsic contextual details. Future research should extend these findings to larger, older adult samples utilizing paradigms that minimize task-relevant support and vary the intrinsic/extrinsic nature of to be remembered details in order to tease apart the nuances of context memory and metamemory performance.
